# Relationship Between Functional Exercise Capacity and Lung Functions in Obese Chidren

**DOI:** 10.4274/jcrpe.1990

**Published:** 2015-08-31

**Authors:** İlker Tolga Özgen, Erkan Çakır, Emel Torun, Alper Güleş, Merve Nur Hepokur, Yaşar Cesur

**Affiliations:** 1 Bezmialem Vakıf University Faculty of Medicine, Department of Pediatrics, Division of Pediatric Endocrinology, İstanbul, Turkey; 2 Bezmialem Vakıf University Faculty of Medicine, Department of Pediatrics, Division of Pediatric Pulmonology, İstanbul, Turkey; 3 Bezmialem Vakıf University Faculty of Medicine, Department of Pediatrics, İstanbul, Turkey

**Keywords:** Exercise capacity, pulmonary functions, obesity

## Abstract

**Objective:**

Cardiovascular, respiratory and musculoskeletal system disorders which may affect the functional exercise capacity are common in obese patients. We aimed to investigate the functional exercise capacity and its relationship with functional pulmonary capacity in obese children.

**Methods:**

A total of 74 obese and 36 healthy children as a control group were enrolled in the study. Pulmonary functions and functional exercise capacity were measured by spirometry and six-minute walk test (6 MWT), respectively.

**Results:**

The distances covered during the 6 MWT in obese and control groups were 570.9±67.5 and 607.8±72.5 meters, respectively (p=0.010). In spirometric pulmonary function tests (PFTs), forced expiratory volume in 1 sec (FEV1) and forced mid-expiratory flows (25-75) were lower in the obese group (p=0.048 and p=0.047, respectively), whereas forced vital capacity (FVC), the FEV1/FVC ratio and peak expiratory flow were not statistically different between the obese and control groups. Multiple regression analysis revealed that among all parameters of anthropometric measures and PFTs, only body mass index standard deviation score (BMI-SDS) was the independent factor influencing 6 MWT.

**Conclusion:**

Functional exercise and lung capacities of obese children were diminished as compared to those of non-obese children. The most important factor influencing functional exercise capacity was BMI-SDS.

## INTRODUCTION

Worldwide childhood overweight and obesity are estimated to be as high as 43 million and this prevalence continues to increase each year (1). Obesity is associated with systemic dysfunctions such as morbidity in cardiovascular, musculoskeletal and respiratory systems even in childhood (2,3,4). All disorders in these systems may also influence the functional exercise capacity.

Childhood obesity has been suggested to be associated with impairment of pulmonary functions and also with asthma. Previous studies have demonstrated that increased weight status in children and adolescents is associated with a general reduction in lung volume measurements, which may reflect impaired lung function, decreased functional status and increased respiratory symptoms (4,5).

The six-minute walk test (6 MWT) is a practical useful test for evaluating the functional exercise capacity. Walking is an activity performed daily even by severely impaired patients. This test measures the distance (D) that a patient can quickly walk on a flat, hard surface within a period of 6 minutes (6 MWD). The global and integrated responses of all the systems involved during exercise, including the pulmonary, cardiovascular and neuromuscular systems may be evaluated with this simple test. The 6 MWT assesses the submaximal level of functional capacity. Most patients choose their own intensity of exercise and are allowed to rest. Also, they do not achieve their maximal exercise capacity during the 6 MWT. However, the 6 MWD is useful in that it may better reflect the functional exercise level of the patient for daily physical activities because most activities of daily living are performed at submaximal levels of exertion (6).

The 6 MWT will not provide specific information on the function of each different organ and system involved in the exercise or on the mechanism of exercise limitation. There are a few studies demonstrating the relationship between spirometric parameters and 6 MWD. We suggested that impaired pulmonary functions may be associated with reduced functional exercise capacity. Therefore, we investigated the pulmonary function tests (PFTs) and functional exercise capacity in obese children and their interrelationships.

## METHODS

In this study, we enrolled a total of 74 (31 males, 43 females) obese children with a mean age of 13.4±2.3 years and 36 (17 males, 19 females) non-obese children with a mean age of 12.7±1.9 years serving as a control group. This study has been approved by the local ethics committee (the decision date and number: 08.08.2012-21/17). Standing height was measured to the nearest 0.1 cm with a Harpenden fixed stadiometer and body weight was measured on a SECA balance scale to the nearest 0.1 kg, with subjects dressed in a light T-shirt and shorts. Obesity was defined according to the body mass index (BMI) >95th percentile using the definition of the International Task Force of Obesity in Childhood and population-specific data (7,8). Obese children did not differ significantly from normal-weight children in age, gender and pubertal stage. All subjects underwent a detailed physical examination including evaluation for syndromes and endocrine diseases as well as a laboratory evaluation including thyroid function tests and diurnal variation of cortisol. Children with syndromal (Laurence-Moon-Biedl, Prader-Willi syndromes, etc) and endocrine conditions (Cushing’s syndrome, hypothyroidism, etc) accompanied by obesity were excluded. Patients who had a history or evidence of metabolic, cardiovascular, respiratory or hepatic disease were excluded.

Hematocrit levels of the obese and control groups were recorded. Plasma insulin was measured by the electrochemiluminescence immunoassay method using an automated immunoassay analyzer (E170, Roche, Hitachi, Osaka, Japan). Glucose measurements were carried out with photometric hexokinase method by using Advia 1800 chemistry analyser (Siemens Healthcare Diagnostics, IL, USA). Homeostasis model assessment of insulin resistance (HOMA-IR) index (fasting insulin x fasting glucose/22.5) was used to determine IR (9). Insulin resistance criteria were HOMA-IR>4.0 for adolescents and HOMA-IR>2.5 for prepubertal children (10).

Spirometric PFTs such as forced vital capacity (FVC), forced expiratory volume in 1 sec (FEV1), FEV1/FVC ratio, forced mid-expiratory flows [FEF (25-75)] and peak expiratory flow (PEF) were performed both in control and obese groups (Spirolab III, MIR®, Rome, Italy). The 6 MWT was applied in accordance with the guidelines approved by the American Thoracic Society (6). The 6 MWT test was performed indoors in a rectangular space with a hard surface. The walking course was 30 m in length. The subjects were asked to wear comfortable clothing and appropriate shoes and to continue their usual medical regimen. They were allowed to have a light meal before early morning or early afternoon tests. Every step of the measurements for the 6 MWT was given below.

a. Repeat testing was performed at about the same time of the day to minimize intraday variability.

b. A “warm-up” period before the test was not performed.

c. The patient was made to wait for at least 10 minutes before the test sitting in a chair located close to the starting point. During this resting time, pulse, blood pressure and oxygen saturation (SpO2) were measured.

d. The Borg scale was used to assess and rate the baseline and postwalk dyspnea and overall fatigue score (6).

e. At the end of the test, the 6 MWD observationns were recorded for walking distance as meters. Pulse, blood pressure and SpO2 values of the subjects were also recorded.

### Statistical Analysis

All statistics were performed using the program SPSS 16.0 for Windows. We used student’s t-test to compare the laboratory and clinical data of the two groups. Pearson’s correlation test was used for evaluation of the relationships of BMI standard deviation score (BMI-SDS), HOMA-IR, hematocrit, height, arterial tension and PFTs with 6 MWD. These parameters were also investigated using multiple regression analysis to assess the predictors of 6 MWD.

## RESULTS

The demographic, clinical and laboratory features of obese and control groups are given in Table 1. The 6 MWD was shorter in the obese group as compared to the controls (570.9±67.5 meters versus 607.8±72.5 meters, respectively; p=0.010). In postwalk assessment, lean subjects had higher dyspnea scores than the obese group (p<0.001). In spirometric tests, the FEV1 and FEF (25-75) were lower in the obese group than controls, whereas there were no differences between the two groups regarding FVC, FEV1/FVC and PEF.

Bivariate correlation analyses revealed that there was a negative correlation only between 6 MWD and BMI-SDS (r=-0.357, p<0.001) and that the 6 MWD did not correlate with other parameters such as HOMA-IR, hematocrit, height, arterial blood pressure and PFTs (Table 2).

Multiple regression analyses demonstrated that among all parameters (BMI-SDS, HOMA-IR, hematocrit, height, arterial blood pressure, PFTs), only BMI-SDS was the predictor of 6 MWD (β=-0.299, p<0.001).

## DISCUSSION

In previous studies, both on adults and children, it has been reported that the 6 MWT showed good reproducibility and known group validity and that it could be recommended for evaluating walking ability in obese subjects. The same studies have also demonstrated that obese patients have a lower exercise capacity than lean subjects (11,12). Morinder et al (12) have reported that the 6 MWD performed by obese children averaged 86% of the distance normal-weight children walked. In adults, slower fast gait speeds with correspondingly shorter stride lengths, poorer sit-to-stand performance and endurance was also associated with obesity (13). In our study, we also found that obese children had lower functional exercise capacity, but the cause of this dysfunction was not clear. Interestingly, obese subjects had a lower dyspnea score in postwalk assessment, an unexpected finding. Morinder et al (12) have also reported a lower heart rate after the 6 MWT in the obese group and they have speculated that motivation and attitude towards physical activity might be the cause of the lower rate in the obese group. We also interpret this situation as the obese subjects not exerting themselves during the test as hard as the lean subjects and we speculated that this condition could be one of the explanations to the insufficient performance of obese individuals in 6 MWT.

On the other hand, we supposed that the cause of this decrease in functional exercise capacity could be due to impairment in pulmonary functions. Obesity has significant effects upon the pulmonary mechanics. Reduction in chest wall compliance, the relationship of the degree of airways resistance and work of breathing with BMI, early airway closure and resultant gas trapping causing ventilation-perfusion mismatching and subsequent hypoxia due to the reductions in functional residual capacity (FRC) and expiratory reserve volume (ERV), a restrictive defect due to the mass loading on the chest wall and finally, expiratory flow limitation due to early airway closure with the generation of intrinsic positive end-expiratory pressure have been demonstrated in obese adults (14). All these mechanisms cause an increased work of breathing in obese patients. In obese adults, it has been reported that pulmonary functions, especially the expiratory reserve volume and FRC may be affected (15). Obesity has negative impacts on lung function also in children and adolescents. Davidson et al (5) have reported that obesity in children and adolescents is associated with a general reduction in lung volume measurements, which may reflect impaired lung function, increased respiratory symptoms and decreased functional status. Baek et al. have demonstrated that obese children without asthma had significant reductions in baseline FEV1 and FEV1/FVC compared with healthy controls (16). Limited studies in children have failed to demonstrate a relationship between obesity and pulmonary functions (17,18,19). In our study, we found that FEV1 and FEF (25-75) were slightly lower in the obese group, but that there was no statistically significant difference between the groups regarding FEV1/FVC ratio.

There are a few studies demonstrating the relationship between PFTs and exercise capacity (20,21). Gontijo et al (20) have reported a positive correlation between PEF and 6 MWD in obese subjects and have also concluded that subjects who had the higher PEF had the higher physico-functional capacity and consequently, the greater distance covered. Another study has demonstrated that obese asthmatics had decreased functional exercise capacity as they covered the shortest 6 MWD when compared to adolescents with obesity or asthma alone or with neither condition. However, these same authors reported that they could not find any relationship between PFTs and 6 MWT in obese asthmatic subjects (21). In our study, although, our patients had some impairment in spirometric PFTs and shorter 6 MWD, there was no correlation between these parameters and the BMI was the only predictor for 6 MWD in multiple regression analysis.

Another potential risk factor in obese patients for inadequate 6 MWT performances was IR. Individuals with IR and/or type 2 diabetes mellitus manifest decreased maximal oxygen consumption and/or submaximal exercise capacity. In addition, lower VO2 peak and slowed VO2 kinetics have been found to correlate with decreased insulin sensitivity as measured using the euglycemic hyperinsulinemic clamp tests (22), but in our study, we could not find any relation between HOMA-IR and 6 MWD.

In conclusion, this study showed that functional exercise and lung capacities of the obese children were lower than those of the non-obese children. However, a relationship between PFTs and functional exercise capacity was not found. The most important factor influencing functional exercise capacity was BMI-SDS.

## Figures and Tables

**Table 1:**
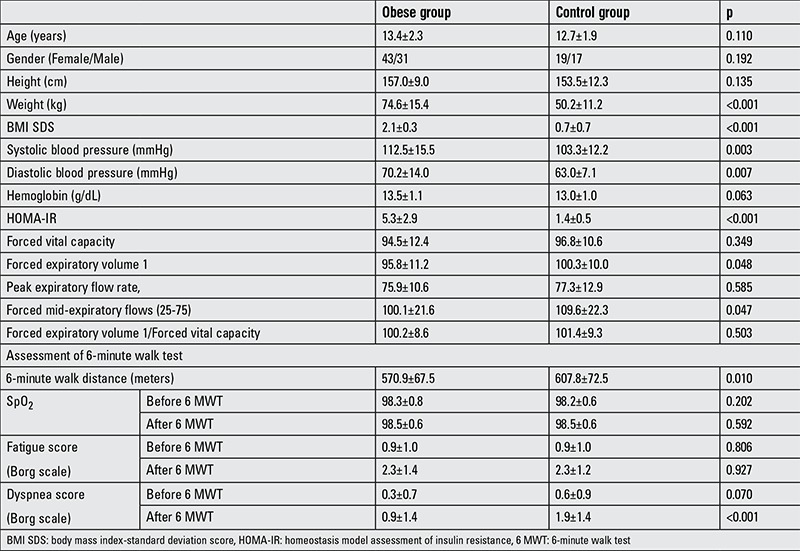
Demographic, clinical and laboratory features of the two groups

**Table 2:**
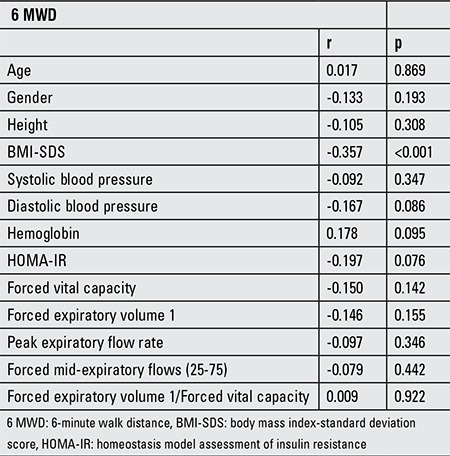
Correlations of 6 MWD with clinical, anthropometric and laboratory measurements

## References

[1] Pulgarón ER (2013). Childhood obesity: a review of increased risk for physical and psychological comorbidities. Clin Ther.

[2] Herouvi D, Karanasios E, Karayianni C, Karavanaki K (2013). Cardiovascular disease in childhood: the role of obesity. Eur J Pediatr.

[3] Adams AL, Kessler JI, Deramerian K, Smith N, Black MH, Porter AH, Jacobsen SJ, Koebnick C (2013). Associations between childhood obesity and upper and lower extremity injuries. Inj Prev.

[4] He QQ, Wong TW, Du L, Jiang ZQ, Qiu H, Gao Y, Liu JW, Wu JG, Yu IT (2009). Respiratory health in overweight and obese Chinese children. Pediatr Pulmonol.

[5] Davidson WJ, Mackenzie-Rife KA, Witmans MB, Montgomery MD, Ball GD, Egbogah S, Eves ND (2014). Obesity negatively impacts lung function in children and adolescents. Pediatr Pulmonol.

[6] ATS Committee on Proficiency Standards for Clinical Pulmonary Function Laboratories (2002). ATS statement: guidelines for the six-minute walk test. Am J Respir Crit Care Med.

[7] Bundak R, Furman A, Gunoz H, Darendeliler F, Bas F, Neyzi O (2006). Body mass index references for Turkish children. Acta Paediatr.

[8] Cole TJ, Bellizzi MC, Flegal KM, Dietz WH (2000). Establishing a standard definition for child overweight and obesity worldwide:international survey. BMJ.

[9] Matthews DR, Hosker JP, Rudenski AS, Naylor BA, Treacher DF, Turner RC (1985). Homeostasis model assessment: insulin resistance and b-cell function from fasting plasma glucose and insulin concentrations in man. Diabetologia.

[10] Valerio G, Licenziati MR, Iannuzzi A, Franzese A, Siani P, Riccardi G, Rubba P (2006). Insulin resistance and impaired glucose tolerance in obese children and adolescents from Southern Italy. Nutr Metab Cardiovasc Dis.

[11] Larsson UE, Reynisdottir S (2008). The six-minute walk test in outpatients with obesity: reproducibility and known group validity. Physiother Res Int.

[12] Morinder G, Mattsson E, Sollander C, Marcus C, Larsson UE (2009). Six-minute walk test in obese children and adolescents: reproducibility and validity. Physiother Res Int.

[13] Pataky Z, Armand S, Müller-Pinget S, Golay A, Allet L (2014). Effects of obesity on functional capacity. Obesity (Silver Spring).

[14] Mandal S, Hart N (2012). Respiratory complications of obesity. Clin Med.

[15] Jensen ME, Wood LG, Gibson PG (2012). Obesity and childhood asthma-mechanisms and manifestations. Curr Opin Allergy Clin Immunol.

[16] Baek HS, Kim YD, Shin JH, Kim JH, Oh JW, Lee HB (2011). Serum leptin and adiponectin levels correlate with exercise-induced bronchoconstriction in children with asthma. Ann Allergy Asthma Immunol.

[17] Boran P, Tokuc G, Pisgin B, Oktem S, Yegin Z, Bostan O (2007). Impact of obesity on ventilatory function. J Pediatr (Rio J).

[18] Peters JI, McKinney JM, Smith B, Wood P, Forkner E, Galbreath AD (2011). Impact of obesity in asthma: evidence from a large prospective disease management study. Ann Allergy Asthma Immunol.

[19] Consilvio NP, Pillo S, Verini M, Giorgis T, Cingolani A, Chiavaroli V, Chiarelli F, Mohn A (2010). The reciprocal influences of asthma and obesity on lung function testing, AHR, and airway inflammation in prepubertal children. Pediatr Pulmonol.

[20] Gontijo PL, Lima TP, Costa TR, Reis EP, Cardoso FP, Cavalcanti Neto FF (2011). Correlation of spirometry with the six-minute walk test in eutrophic and obese individuals. Rev Assoc Med Bras.

[21] Rastogi D, Khan UI, Isasi CR, Coupey SM (2012). Associations of obesity and asthma with functional exercise capacity in urban minority adolescents. Pediatr Pulmonol.

[22] Reusch JE, Bridenstine M, Regensteiner JG (2013). Type 2 diabetes mellitus and exercise impairment. Rev Endocr Metab Disord.

